# Thyroid Hormone Neuroprotection Against Perfluorooctane Sulfonic Acid Cholinergic and Glutamatergic Disruption and Neurodegeneration Induction

**DOI:** 10.3390/biomedicines12112441

**Published:** 2024-10-24

**Authors:** Paula Moyano, Gabriela Guzmán, Andrea Flores, Jimena García, Lucia Guerra-Menéndez, Javier Sanjuan, José Carlos Plaza, Luisa Abascal, Olga Mateo, Javier Del Pino

**Affiliations:** 1 Department of Pharmacology and Toxicology, Veterinary School, Complutense University of Madrid, 28040 Madrid, Spain; 2 Departamento de Ciencias Meìdicas Baìsicas, Facultad de Medicina, Universidad San Pablo-CEU, Urbanizacioìn Montepriìncipe, 28660 Boadilla del Monte, Spain; 3 Department of Legal Medicine, Psychiatry and Pathology, Medicine School, Complutense University of Madrid, 28040 Madrid, Spain; 4 Department of Surgery, Medicine School, Complutense University of Madrid, 28040 Madrid, Spain

**Keywords:** perfluorooctane sulfonic acid, thyroid hormones, basal forebrain, cholinergic neurons, glutamatergic neurotransmission, AChE, cholinergic neurotransmission, neurodegeneration

## Abstract

**Background**: Perfluorooctane sulfonic acid (PFOS), a widely used industrial chemical, was reported to induce memory and learning process dysfunction. Some studies tried to reveal the mechanisms that mediate these effects, but how they are produced is still unknown. Basal forebrain cholinergic neurons (BFCN) maintain cognitive function and their selective neurodegeneration induces cognitive decline, as observed in Alzheimer’s disease. PFOS was reported to disrupt cholinergic and glutamatergic transmissions and thyroid hormone action, which regulate cognitive processes and maintain BFCN viability. **Objective/Methods**: To evaluate PFOS neurodegenerative effects on BFCN and the mechanisms that mediate them, SN56 cells (a neuroblastoma cholinergic cell line from the basal forebrain) were treated with PFOS (0.1 µM to 40 µM) with or without thyroxine (T3; 15 nM), MK-801 (20 µM) or acetylcholine (ACh; 10 µM). **Results**: In the present study, we found that PFOS treatment (1 or 14 days) decreased thyroid receptor α (TRα) activity by decreasing its protein levels and increased T3 metabolism through increased deiodinase 3 (D3) levels. Further, we observed that PFOS treatment disrupted cholinergic transmission by decreasing ACh content through decreased choline acetyltransferase (ChAT) activity and protein levels and through decreasing muscarinic receptor 1 (M1R) binding and protein levels. PFOS also disrupted glutamatergic transmission by decreasing glutamate content through increased glutaminase activity and protein levels and through decreasing N-methyl-D-aspartate receptor subunit 1 (NMDAR1); effects mediated through M1R disruption. All these effects were mediated through decreased T3 activity and T3 supplementation partially restored to the normal state. **Conclusions**: These findings may assist in understanding how PFOS induces neurodegeneration, and the mechanisms involved, especially in BFCN, to explain the process that could lead to cognitive dysfunction and provide new therapeutic tools to treat and prevent its neurotoxic effects.

## 1. Introduction

Perfluorooctane sulfonic acid (PFOS), the most used of perfluorinated compounds, has been extensively employed due to its properties in industrial (coatings, fire foam, leaning agents, textiles, lubricants, among others) and commercial applications (leather, textiles, furniture carpets, among others) [1,2,3]. PFOS was declared a persistent organic compound and banned from Europe and the United States, but it remained being used in other countries, and together with its persistence, it continues to be present in the environment [4] and detected in wildlife and human biological fluids [5]. Different toxic effects were described including cardiovascular toxicity, immunotoxicity, endocrine disruption, reproductive and developmental toxicity, hepatotoxicity, and neurotoxicity among other toxic effects [1]. Serum PFOS levels were correlated with cognitive dysfunction in humans [6], and human brain accumulation was associated with Alzheimer’s disease (AD) [7]. PFOS was also described as inducing memory and learning process disruption in animals [8,9], but the mechanisms through which this effect is mediated are not well understood.

The central cholinergic system (CCS) regulates cognitive functions [10]. The CCS is mainly formed by basal forebrain (BF) cholinergic neurons, which project extensively to the prefrontal cortex and hippocampus, regulating cognitive functions [10,11]. CCS integrity, both cholinergic neurotransmission and/or BF cholinergic neurons (BFCN) innervation, is necessary to maintain cognitive functions, and cholinergic transmission disruption or BFCN loss, as reported in AD, leads to hippocampal and frontal cortex neurodegeneration and cognitive dysfunction [10,12]. PFOS studies on cognitive function related this alteration to the observed apoptotic neuronal cell death in the frontal cortex and hippocampal neurons of animals [8,9]. PFOS was reported to induce neurodegeneration of cholinergic neurons in the nematode Caenorhabditis elegans [13]. However, the possible BFCN neurodegeneration induction by PFOS as a feasible cause of hippocampus and frontal cortex damage and cognitive dysfunction observed was not explored. Therefore, it is necessary to explore the possible damage of cholinergic neurotransmission and/or BFCN loss produced by PFOS exposure as a reasonable cause of the cognitive decline reported.

PFOS was reported to alter cholinergic transmission, disrupting acetylcholine (ACh) levels [14,15], acetylcholinesterase (AChE) activity [16,17,18,19], choline acetyltransferase (ChAT) activity and expression [17,18,20], and muscarinic receptors expression [14,16], in different species. ACh regulates cognitive function and participates in the maintenance of cell viability [21], and its disruption could lead to neuronal death and cognitive decline. Muscarinic 1 receptor (M1R) mediates BFCN viability and cognitive function, and its downregulation or blockage, as observed in AD, produces memory impairment [22,23], and BFCN loss [24,25]. AChE is formed in two variants (R and S), which present opposite actions on cell viability maintenance, inducing apoptotic and necrotic cell death when the AChE-S variant is upregulated [26,27], but AChE silencing blocks its apoptotic cell death induction [28]. Therefore, PFOS could disrupt the CCS and/or induce BFCN loss, leading to the cognition dysfunction reported after PFOS exposure.

Glutamate, the main central nervous system excitatory neurotransmitter, is necessary to maintain neuronal viability and cognitive functions [29,30,31]. However, elevated glutamate levels lead to neurodegeneration and cognitive decline [29,30,31]. Glutamatergic N-methyl-D-aspartate receptors (NMDAR) are necessary to maintain synaptic plasticity, cognitive function, and neuronal viability, but their overactivation leads to cognitive dysfunction and neurodegeneration [30]. The cholinergic system regulates glutamate neurotransmission [32], and M1R was shown to regulate glutamate levels [33,34], and NMDAR expression and activity [35]. PFOS was shown to increase glutamate levels and induce excitotoxicity in rat primary cerebellar granule neurons through activation of NMDAR following 24 h of treatment [36]. PFOS also was shown to increase glutamate levels in mouse hippocampal neurons after repeated exposure, relating this effect to the apoptotic neurodegeneration and cognitive decline observed in these animals [37,38]. Thus, PFOS could induce excitotoxicity through cholinergic transmission disruption mediated by M1R dysfunction, leading to neurodegeneration and cognitive decline.

Thyroid hormones (THs) regulate cognitive functions [39,40] and are necessary to maintain BFCN viability [24,41,42]. TH level reduction was associated with BFCN neurodegeneration [24,42,43] and cognitive dysfunction [44]. THs regulate the cholinergic system [45] and were reported to increase ACh metabolism by inducing AChE activity and to induce ACh release [24,46]. THs regulate muscarinic receptor ligand affinity and expression of AChE variants, muscarinic receptors, and ChAT [24,47,48]. Further, THs control glutamatergic transmission, regulating glutamate levels [49,50], mediating glutamate release and glutaminase activity [51], and regulating MNDAR expression [52]. PFOS was reported to decrease TH levels in animals and humans, through altering their synthesis and metabolisms [5]. PFOS alters the activity of deiodinase enzymes and was reported to upregulate Deiodinase 3 (D3) expression after repeated exposure in adult male rat liver [53]. D3 is the main TH-metabolizing enzyme expressed in neurons [54], which metabolizes triiodothyronine (T3) to diiodothyronine (T2) and tetraiodothyronine (T4) to reverse T3, and its upregulation decreases T3 levels [5]. PFOS also disrupts thyroid hormone receptor (TR) pathways by binding to TR alpha (TRα) [55], which is the main TR type expressed in the brain [56], antagonizing them. Therefore, PFOS may disrupt cholinergic and glutamatergic transition, leading to BFCN loss through TH activity disruption, thus leading to cognitive decline.

According to the observations expressed above, we hypothesized that PFOS could disrupt TH activity, triggering cholinergic transmission disruption that mediates glutamatergic transmission alteration and finally inducing BFCN cell death following single and repeated treatment. Therefore, the present study aimed to prove this hypothesis in a model of BFCN, to provide a new understanding of the neurotoxic mechanisms that could produce neurodegeneration in BFCN and lead to cognitive decline to provide new therapeutic tools to treat these toxic effects.

## 2. Materials and Methods

### 2.1. Chemicals

Sigma (Madrid, Spain) provided the perfluorooctane sulfonate (≥99%), acetylcholine, acetylthiocholine, tetraisopropylpyrophosphoramide (iso-OMPA), dimethyl sulfoxide (DMSO), dithionitrobenzoic acid, dibutyryl-cAMP, MK-801, poly-l-lysine, retinoic acid, 3-[4,5-dimethylthiazol-2-yl]-2,5-diphenyl-tetrazolium bromide (MTT), and triiodothyronine. All other chemicals were reagent grade of the highest laboratory purity available.

### 2.2. SN56 Culture Procedure

Professor Laura Calzà from CIRI-SDV and Fabit at the University of Bologna kindly gifted us the murine cell line from the BFCN, SN56 [57], which we used as a BF cholinergic neurons’ model to study the deleterious effects of PFOS induced on cholinergic neurons and the mechanisms through which they are mediated. Cells were cultured following the procedure established by Moyano et al. [26]. We discarded the culture medium and added a freshly made one every 48 h [58]. To obtain cells more sensitive to cholinergic neurotoxicity, SN56 cell cultures were differentiated following the protocol described in the literature [59,60]. Cells were checked to be mycoplasma-free using the Sigma (Madrid, Spain) Look Out mycoplasma kit.

To determine glutamate and ACh content, ChAT, glutaminase, M1R, TRα, NMDAR1, and D3 protein levels, glutaminase, ChAT, and AChE activity, TRα activity, *glutaminase*, *Ache*, *M1r*, *Ache-S*, and *Ache-R* gene expression, *M1r* or *Ache* silencing effects, cell viability, and caspases 3/7 activation, we seeded cells previously differentiated (passages 7–15) in 6-well plates at a density per well of 2 × 10^6^ or 10^6^ (1- or 14-day exposure, respectively) or 96-well plates at a density per well of 4000 or 2000 (1- or 14-day exposure, respectively) in DMEM medium and treated with vehicle or PFOS (0.1 µM to 40 µM) either for 24 h or 14 days and including or not the NMDAR1 antagonist MK-801 (20 µM) and/or T3 (15 nM), and/or ACh (10 µM). With each medium change, the treatments were added in the fresh medium. The vehicle chosen was 0.1% dimethyl sulfoxide (DMSO), in which PFOS was dissolved. We carried out a negative control that only contained the vehicle. Data from control groups from each study were not statistically different between 1- and 14-day exposure; therefore, these data have been combined and are shown in only one white bar. Each experimental procedure was performed in at least 3 wells.

PFOS is fast and widely distributed in the body and accumulates in different tissues as the brain [61], presenting an estimated 5.4 years of serum half-lives in humans [5]. It was reported that plasma range concentrations detected were from 0.002 μM to 0.23 μM in the general population and from 0.988 μM to 10 μM in the occupationally exposed population [62]. We chose a range of concentrations for PFOS exposure (0.1 µM to 40 µM) based on the concentrations to which humans might be exposed to and that have been used to elucidate in vitro the PFOS toxic mechanisms [2,3,63]. Lastly, we selected the concentration of 10 µM PFOS to elucidate the possible mechanisms that induce neurodegeneration on SN56 cells following exposure to PFOS for 1 or 14 days, as this concentration has been shown to reduce the viability of cells, TRα activity, and alter both glutamatergic and cholinergic transmission.

### 2.3. Analysis of TRα Activity

TRα activation was determined in nuclear extracts using the Thyroid Hormone Receptor Alpha Transcription Factor Activity (ELISA) Assay Kit (TFAB00173; Assay Genie, Dublin, Ireland), following the manufacturer’s directions. Protein concentrations were determined by the Thermo Fisher Scientific (Madrid, Spain) BCA kit, according to the manufacturer’s procedures. The TRα activation assay is based on the detection of activated TRα present in nuclear extracts, obtained after treatments, through measurement of its selective binding to an immobilized specific double-stranded DNA oligonucleotide sequence that contains the TRα consensus binding site. The detection of bounded TRα is performed first by a primary antibody that recognizes an epitope of it that is accessible only when the protein is activated and bound to its target DNA and then by the secondary antibody conjugated with HRP, which catalyzes the colorimetric reaction after adding TMB (3, 3′, 5, 5′-Tetramethylbenzidine) developing blue color, which absorbance is proportional to target protein concentration in the sample. All samples were read at 450 nm wavelength with a Fluoroskan FL microplate reader (Thermo Fisher, Madrid, Spain). ELISA results were normalized with nuclear protein concentration presented as percent untreated control.

### 2.4. Evaluation of Acetylcholine Content

We measured the concentration of Ach in the culture medium using a commercial colorimetric/fluorimetric kit from Abcam (Cambridge, UK) [64]. The wavelength used to measure the fluorescence was λ Ex/Em 535/587 nm, which was measured with a Fluoroskan FL microplate reader (Thermo Fisher, Madrid, Spain). A standard choline curve was used to plot the fluorescence of every sample, leading to pmol/well units of ACh levels. Results were presented as percent untreated control.

### 2.5. Analysis of AChE Enzymatic Activity

To research the activity of AChE, we used Ellman’s technique [65], including modifications from the literature [66,67], and a normalization with total protein levels. Butyrylcholinesterase activity was inhibited using iso-OMPA. We read the absorbance at 412 nm with a Fluoroskan FL microplate reader (Thermo Fisher, Madrid, Spain). We calculated the results as nmol/h/mg protein and present them as relative to untreated control.

### 2.6. Analysis of ChAT Activity

ACh synthesis is catalyzed by ChAT. We followed the procedure described by Zheng et al. [68] to homogenize the samples from cultures (control and treatments) of 6-well plates for each sample. The Fonnum assay was used to measure the activity of ChAT, including a modification from the literature [69] by incorporating [^14^C] acetyl-CoA into ACh, as described before [68,70]. Units of ChAT activity values are pmol ACh synthesized/h/mg protein and are shown as percent untreated control.

### 2.7. M1R Radioligand Binding Assay

PFOS disruption of [^3^H] pirenzepine (Perkin Elmer, Madrid, Spain) binding to M1R on cell membranes was measured in homogenates of SN56 cells obtained following the protocol described by Del Pino et al. [71]. Binding values obtained (pmol) were normalized by protein concentration values resulting in pmol/mg units and presented as percent untreated control.

### 2.8. Protein Determination

We determined the level of total protein in supernatants homogenized that were obtained following the procedure by Moyano et al. [26] using the Thermo-Fisher Scientific BCA kit (Madrid, Spain).

The quantification of protein levels of ChAT, glutaminase, M1R, TRα, NMDAR1, and D3 proteins was determined with ELISA commercial kits (MBS3806699, MBS2020175, MBS176926, MBS9313355, MBS8807324, and MBS2018962, respectively, MyBioSource, San Diego, CA, USA), following the producers indications. The absorbance was determined at 450 nm with a Fluoroskan FL microplate reader (Thermo Fisher, Madrid, Spain). We performed a negative control by ELISA assay to determine that there were no interferences between PFPS and A normalization by total protein concentration was performed to avoid a bias on the data by the cell death induced. Units of protein levels are ng/mg of protein and are shown as the percentage of the untreated control.

### 2.9. Gene Expression Analysis

To determine gene expression, we followed the procedure by Moyano et al. [26] and evaluated the results following Livak and Schmittgen [72]. The qPCR analysis followed the MIQUE requirements. We used primers, from SA Biosciences, validated for mRNAs encoding *M1r* (PPM03990A), *beta-actin* (*Actb*, housekeeping gene; PPM02945B), *glyceraldehyde-3-phosphate dehydrogenase* (*Gapdh*, housekeeping gene; PPM02946E) and *Ache* (PPM35356A) together with the Real-Time SYBR Green PCR master mix (SA Biosciences, PA-012) to run qPCR in a CFX96. Primers used for *Ache-R* and *Ache-S* are shown in Table 1 [73]. The negative control performed was a qPCR that did not include cDNA.

### 2.10. Glutamate Content

After the last PFOS treatment, we used cell lysates and culture medium to quantify glutamate content following the procedures detailed in the literature [32]. We followed procedures established in previous studies to process samples before they were analyzed by HPLC [74,75,76,77]. Results were normalized with total protein levels obtained using BCA kit and units obtained were μmol/mg of protein and data are shown as the percentage relative to controls.

### 2.11. Glutaminase Function

We followed the instructions on the Glutaminase Microplate Assay Kit (MBS8243221, MyBioSource, San Diego, CA, USA), and the literature procedures [32], to determine the activity of glutaminase. Normalization by total protein levels obtained using a BCA kit was performed and resulted in μmol/h/mg protein units, which are shown as percentages relative to controls.

### 2.12. AChE and M1R Silencing

The procedure by Moyano et al. [26] was followed in order to silence *Ache* and *M1r* We obtained from Qiagen (Barcelona, Spain) two siRNA duplex sets homologous to the sequences for mouse *Ache* and *M1r* (GS11423, and GS12669, respectively). The transfection control used was the All Stars Negative Control siRNA (Qiagen, Barcelona, Spain). Once 48 h had passed from the silencing procedure, we analyzed the efficacy of the silencing of *Ache* and *M1R* determining the expression of both genes by qPCR, and we observed a decrease in the expression of both genes that was statistically significant. Treatment with PFOS was performed, as scheduled, once 24 h of transfection had passed, after washing the cultures with PBS.

### 2.13. Evaluation of Cell Viability and Activation of Caspases 3/7

We used MTT assay to evaluate the effect of PFOS treatment on the viability of SN56 cells. We incubated cells during 4 h with MTT yellow reagent (100 µL, 0.5 mg/mL pattern solution) at 37 °C. We added 250 µL of DMSO after discarding the culture medium after incubation, which solubilizes the purple formazan that has been precipitated. This solution was read at 570 nm with a Fluoroskan FL microplate reader (Thermo Fisher, Madrid, Spain). DMSO treatment was used as a control. The results obtained were expressed as a percentage relative to the controls.

Caspase activation was evaluated with Caspase-Glo 3/7 luminescence assay kit (G8090, Promega, Madrid, Spain), as a marker of apoptosis, following the manufacturer’s guidelines. We used a Perkin Elmer LS50B plate-reading luminometer (Madrid, Spain) to determine the luminescence of each sample. Data are shown as percentages relative to controls.

### 2.14. Statistical Analysis

Results are shown as the mean ± standard error of the mean (SEM) and represent the data of three replicates of cultures performed three different times. Firstly, data normality and variance homogeneity were corroborated by performing Shapiro–Wilk’s and Levene’s tests, respectively. We compared single treatments/transfections using Student’s *t*-test and used one-way or two-way ANOVA analyses, and the Tukey post hoc test, to compare treatments with response or transfections with treatments, respectively and observe whether there were differences that were statistically significant (*p* ≤ 0.05), through the GraphPad 5.0 Software Inc.’s (San Diego, CA, USA).

## 3. Results

### 3.1. Analysis of TRα and D3 Protein Content and TRα Activity

**Figure 1:**
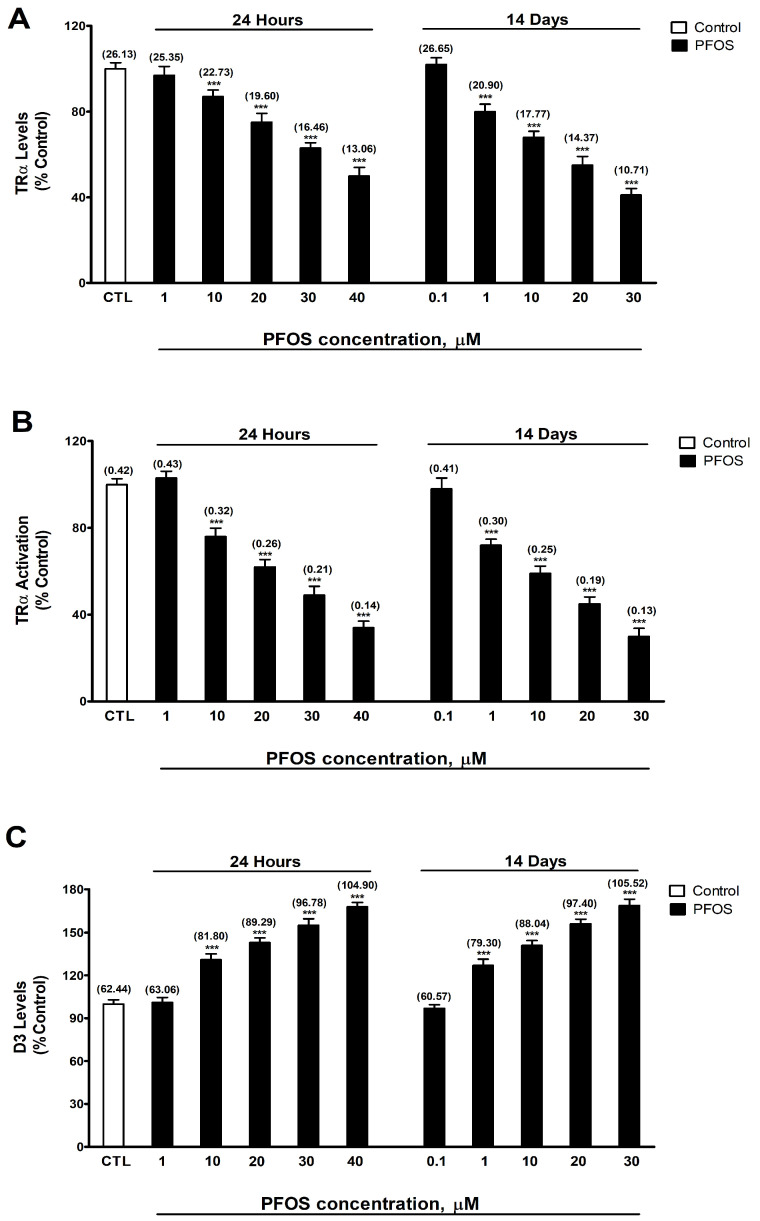
(**A**) TRα levels, (**B**) TRα activation, and (**C**) D3 levels. The mean ± SEM was obtained from data of three replicates of cultures performed three different times. Results are shown as percentages relative to controls taken as 100%. Data within parentheses above the bars represent absolute values of TRα and D3 protein concentrations (ng/mg) and TRα activity (absorbance at 450 nm). One-way (PFOS concentrations-response comparisons) and two-way (time-treatment comparisons) ANOVA analyses followed by the Tukey post hoc test were developed to determine statistically significant differences between treatments. *** *p* ≤ 0.001, significantly different from controls.

TRα and D3 protein content and TRα activity were determined after one- and fourteen-day PFOS treatment (0.1–40 µM) in SN56 cells. Two-way ANOVA analysis showed a significant time versus PFOS treatment interaction following single and repeated PFOS treatment in TRα activity (F_(6,112)_ = 39.5, *p* < 0.0001), TRα (F_(6,112)_ = 37.2, *p* < 0.0001), and D3 (F_(6,112)_ = 29.2, *p* < 0.0001) protein content. One-way ANOVA analysis showed a significant concentration-dependent PFOS treatment effect following single (TRα levels: F_(6,35)_ = 287.9, *p* < 0.0001; and D3 levels: F_(6,35)_ = 544.9, *p* < 0.0001; and TRα activity: F_(6,35)_ = 597.1, *p* < 0.0001) and repeated (TRα levels: F(_6,35_) = 498.9, *p* < 0.0001; and D3 levels: F(_6,35_) = 635.9, *p* < 0.0001; and TRα activity: F(_6,35_) = 572.5, *p* < 0.0001) treatment. PFOS statistically significantly increased TRα protein content (Figure 1A) and TRα activity (Figure 1B), but statistically significantly decreased D3 protein content (Figure 1B) after one (starting at 10 μM concentration) and fourteen days (starting at 1 μM concentration) of exposure, and these alterations increased following the treatment concentration level. We did not observe a statistically significant difference between controls to which vehicle was added and those in which it was not. This was performed to corroborate that vehicle has no effect on the results.

### 3.2. Analysis of Cholinergic Neurotransmission (ACh Content, AChE Activity, AChE-S/R Gene Expression, ChAT Activity and Protein Content, and M1R Protein Content and Radioligand Binding Analysis)

**Figure 2:**
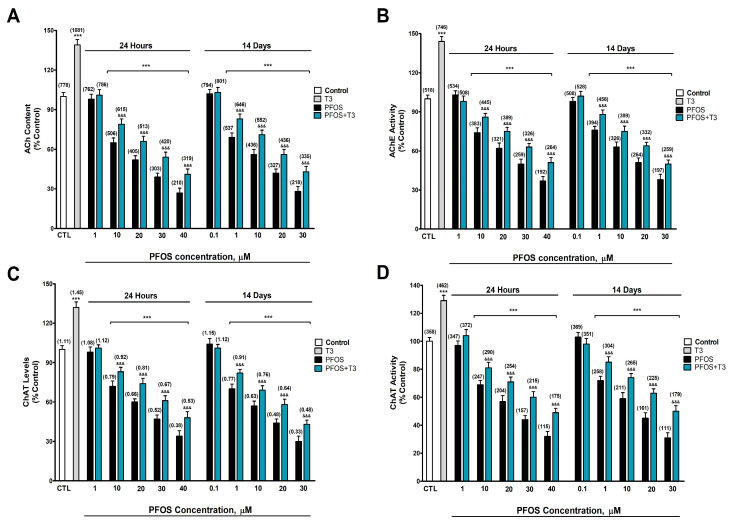
(**A**) Acetylcholine (ACh) content, (**B**) acetylcholinesterase (AChE) activity, (**C**) choline acetyltransferase (ChAT) levels, and (**D**) ChAT activity. The mean ± SEM was obtained from data of three replicates of cultures performed three different times. Results are shown as percentages relative to controls taken as 100%. Data within parentheses above the bars represent absolute values of ACh concentrations (pmol/mL), AChE activity (nmol/h/mg), ChAT levels (ng/mg) and activity (pmol/h/mg). Student’s *t*-test (T3 treatment-control or PFOS + T3 treatment-PFOS treatment to each PFOS concentration comparisons), one-way (PFOS concentrations-response comparisons) and two-way (time-treatment comparisons) ANOVA analyses followed by the Tukey post hoc test were developed to determine statistically significant differences between treatments. *** *p* ≤ 0.001, significantly different from controls; ^&&&^ *p* ≤ 0.001 compared with PFOS treatment.

ACh content, AChE activity, and ChAT activity and protein content were determined after one- and fourteen-day PFOS treatment (0.1–40 µM) in SN56 cells. Two-way ANOVA analysis showed a significant time versus PFOS treatment interaction following single and repeated PFOS treatment in ACh content (F_(6,112)_ = 39.9, *p* < 0.0001), AChE activity (F_(6,112)_ = 37.2, *p* < 0.0001), ChAT levels (F_(6,112)_ = 37.2, *p* < 0.0001), and ChAT activity (F_(6,112)_ = 39.5, *p* < 0.0001) protein content. One-way ANOVA analysis showed a significant concentration-dependent PFOS treatment effect following single (ACh content: F_(6,35)_ = 482.2, *p* < 0.0001; AChE activity: F_(6,35)_ = 468.7, *p* < 0.0001; ChAT levels: F_(6,35)_ = 408.6, *p* < 0.0001; and ChAT activity: F_(6,35)_ = 425.5, *p* < 0.0001) and repeated (ACh content: F_(6,35)_ = 458.5, *p* < 0.0001; AChE activity: F_(6,35)_ = 413.9, *p* < 0.0001; ChAT levels: F_(6,35)_ = 444.1, *p* < 0.0001; and ChAT activity: F_(6,35)_ = 389.5, *p* < 0.0001) treatment. PFOS statistically significantly decreased ACh content (Figure 2A), AChE activity (Figure 2B), ChAT protein content (Figure 2C), and ChAT activity (Figure 2D), compared with the control group, after one (starting at 10 µM concentration) and fourteen days (starting at 1 µM concentration), and these decrements increased following the treatment concentration level. T3 treatment (15 nM) produced a statistically significant increase in ACh content (Figure 2A; t_(10)_ = 18.8, *p* < 0.0001), AChE activity (Figure 2B; t_(10)_ = 22.5, *p* < 0.0001), ChAT protein content (Figure 2C; t_(10)_ = 15.5, *p* < 0.0001), and ChAT activity (Figure 2D; t_(10)_ = 15, *p* < 0.0001) compared with the control group according to Student’s *t*-test statistical analysis. PFOS co-treatment with T3 partially reversed the decrease observed in ACh content (Figure 2A), AChE activity (Figure 2B), ChAT protein content (Figure 2C), and ChAT activity (Figure 2D) after PFOS single and repeated treatment alone (Supplementary Table S1). To confirm that PFOS decrement effects on these targets are linked to their activity and content, and are not related to a decrease in neuron number, we normalized the data with the absolute protein levels, confirming these results.

**Figure 3:**
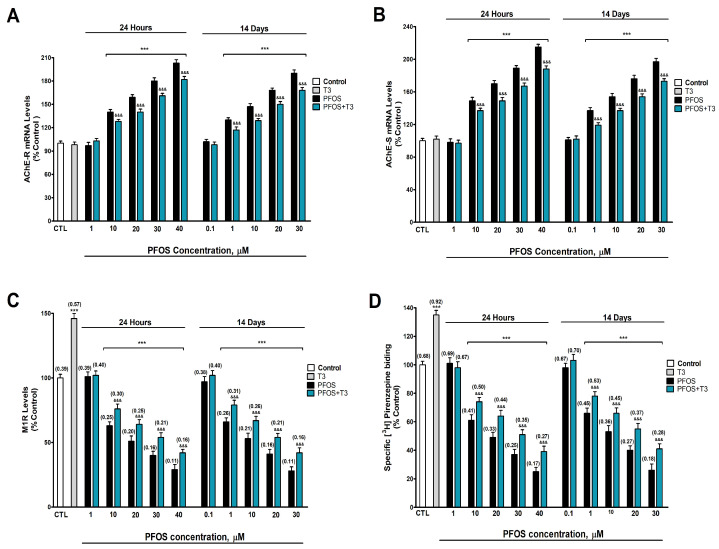
(**A**) AChE-R and (**B**) AChE-S variant gene expression, (**C**) M1R levels, and (**D**) M1R [^3^H] pirenzepine binding. The mean ± SEM was obtained from data of three replicates of cultures performed three different times. Results are shown as percentages relative to controls taken as 100%. Data within parentheses above the bars represent absolute values of M1R levels (ng/mg), and M1R [^3^H] pirenzepine binding (pmol/mg). Student’s *t*-test (T3 treatment-control or PFOS + T3 treatment-PFOS treatment to each PFOS concentration comparisons), one-way (PFOS concentrations-response comparisons), and two-way (time-treatment comparisons) ANOVA analyses followed by the Tukey post hoc test were developed to determine statistically significant differences between treatments. *** *p* ≤ 0.001, significantly different from controls; ^&&&^ *p* ≤ 0.001 compared with PFOS treatment.

AChE-S/R variant gene expression, M1R protein content, and M1R radioligand binding were determined after one- and fourteen-day PFOS treatment (0.1–40 µM) in SN56 cells. Two-way ANOVA analysis showed a significant time versus PFOS treatment interaction following single and repeated PFOS treatment in AChE-R (F_(6,70)_ = 22.1, *p* < 0.0001), and AChE-S (F_(6,70)_ = 51.7, *p* < 0.0001) gene expression, M1R levels (F_(6,70)_ = 31.9, *p* < 0.0001), and M1R radioligand binding (F_(6,70)_ = 26.2, *p* < 0.0001). One-way ANOVA analysis showed a significant concentration-dependent PFOS treatment effect following single (AChE-R gene expression: F_(6,35)_ = 791.1, *p* < 0.0001; AChE-S gene expression: F_(6,35)_ = 1038, *p* < 0.0001; M1R levels: F_(6,35)_ = 544.9, *p* < 0.0001; and M1R radioligand binding: F_(5,48)_ = 597.1, *p* < 0.0001) and repeated (AChE-R gene expression: F_(6,35)_ = 720.5, *p* < 0.0001; AChE-S gene expression: F_(6,35)_ = 494.1, *p* < 0.0001; M1R levels: F_(6,35)_ = 676.8, *p* < 0.0001; and M1R radioligand binding: F_(6,35)_ = 448.4, *p* < 0.0001) treatment. PFOS statistically significantly increased AChE-R (Figure 3A) and AChE-S (Figure 3B) gene expression, but decreased M1R protein content (Figure 3C), and M1R [^3^H] pirenzepine binding (Figure 3D), compared with the control group, after one (starting at 10 µM concentration) and fourteen days (starting at 1 µM concentration), and these increments and decrements increased following the treatment concentration level. T3 treatment (15 nM) produced a statistically significant increase in M1R protein content (Figure 3C; t_(10)_ = 23.9, *p* < 0.0001), and M1R [^3^H]pirenzepine binding (Figure 3D; t_(10)_ = 21.2, *p* < 0.0001) compared with the control group according to Student’s *t*-test statistical analysis. PFOS co-treatment with T3 reversed partially the increase observed in AChE-R (Figure 3A) and AChE-S (Figure 3B) gene expression, and the decrease observed in M1R protein content (Figure 3C), and M1R [^3^H] pirenzepine binding (Figure 3D) after single and repeated PFOS treatment alone (Supplementary Table S2). To confirm that PFOS decrement in M1R protein content, and M1R [^3^H] pirenzepine biding are linked their content and binding, and are not related to a decrease in neurons, we normalized the data with the absolute protein levels, confirming these results.

### 3.3. Analysis of Glutamatergic Transmission (Glutamate Levels, NMDAR1 Protein Levels, and Glutaminase Content and Activity Analysis)

**Figure 4:**
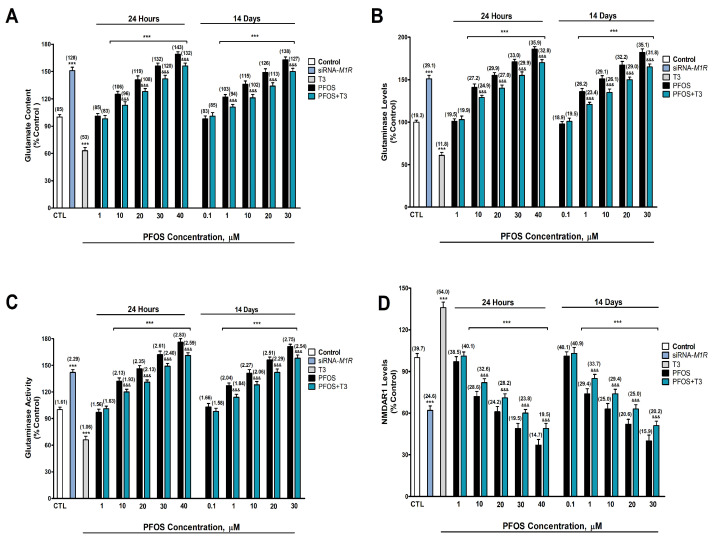
(**A**) Glutamate concentration, (**B**) glutaminase protein content, (**C**) glutaminase activity, and (**D**) NMDAR1 protein content. The mean ± SEM was obtained from data of three replicates of cultures performed three different times. Results are shown as percentages relative to controls taken as 100%. Data within parentheses above the bars represent absolute values of glutamate content (μmol/mg), glutaminase levels (ng/mg) and activity (μmol/h/mg), and NMDAR1 levels (ng/mg). Student’s *t*-test (*siRNA-M1R*-control wild-type cells, T3 treatment-control or PFOS + T3 treatment-PFOS treatment to each PFOS concentration comparisons), one-way (PFOS concentrations-response comparisons), and two-way (time-treatment comparisons) ANOVA analyses followed by the Tukey post hoc test were developed to determine statistically significant differences between treatments. *** *p* ≤ 0.001, significantly different from controls; ^&&&^ *p* ≤ 0.001 compared to PFOS treatment.

Glutamate content, glutaminase and NMDAR1 protein content, and glutaminase activity were determined after one- and fourteen-day PFOS treatment (0.1–40 µM) in SN56 cells. Two-way ANOVA analysis showed a significant time versus PFOS treatment interaction following single and repeated PFOS treatment in glutamate content (F_(6,70)_ = 20.8, *p* < 0.0001), glutaminase levels (F_(6,70)_ = 33.9, *p* < 0.0001), glutaminase activity (F_(6,70)_ = 4.6, *p* < 0.0001), and NMDAR1 levels (F_(6,70)_ = 22.7, *p* < 0.0001). One-way ANOVA analysis showed a significant concentration-dependent PFOS treatment effect following single (glutamate content: F_(6,35)_ = 471.2, *p* < 0.0001; glutaminase levels: F_(6,35)_ = 691.7, *p* < 0.0001; glutaminase activity: F_(6,35)_ = 501.4, *p* < 0.0001; and NMDAR1: F_(6,35)_ = 322.7, *p* < 0.0001) and repeated (glutaminase content: F_(6,35)_ = 388.2, *p* < 0.0001; glutaminase levels: F_(6,35)_ = 528.6, *p* < 0.0001; glutaminase activity: F_(6,35)_ = 442.5, *p* < 0.0001; and NMDAR1: F_(6,35)_ = 289.6, *p* < 0.0001) treatment. PFOS statistically significantly increased glutamate content (Figure 4A), glutaminase protein content (Figure 4B), and glutaminase activity (Figure 4C), but decreased NMDAR1 protein content (Figure 4D), compared with the control group, after one (starting at 10 µM concentration) and fourteen days (starting at 1 µM concentration), and these increments and decrement increased following the treatment concentration level. The *M1r* silencing produced a statistically significant increase in glutamate content (Figure 4A; t_(10)_ = 26.5, *p* < 0.0001), glutaminase protein content (Figure 4B; t_(10)_ = 26.3, *p* < 0.0001), and glutaminase activity (Figure 4C; t_(10)_ = 24.2, *p* < 0.0001), but produced a statistically significant decrease in NMDAR1 protein content (Figure 3D; t_(10)_ = 21.5, *p* < 0.0001) compared with the control group according to Student’s *t*-test statistical analysis. T3 treatment (15 nM) produced a statistically significant decrease in glutamate content (Figure 4A; t_(10)_ = 20.2, *p* < 0.0001), glutaminase protein content (Figure 4B; t_(10)_ = 22.9, *p* < 0.0001), and glutaminase activity (Figure 4C; t_(10)_ = 17.1, *p* < 0.0001), but produced a statistically significant increase in NMDAR1 protein content (Figure 3D; t_(10)_ = 18.1, *p* < 0.0001) compared with the control group according to Student’s *t*-test statistical analysis. PFOS co-treatment with T3 partially reversed the increase observed in glutamate content (Figure 4A), glutaminase content (Figure 4B), and glutaminase activity (Figure 3C), and the decrease observed in NMDAR1 protein content (Figure 3D) after single and repeated PFOS alone treatment (Supplementary Table S3).

### 3.4. Gene Knockdown Evaluation

**Figure 5:**
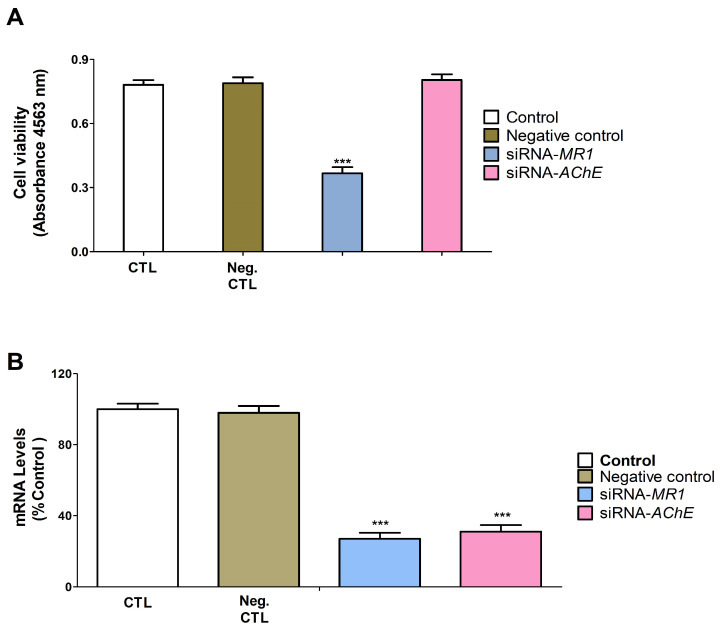
*Ache* or *M1r* knockdown efficiency and effect on neuronal viability. Positive (transfection without siRNA) and negative control (transfection with scrambled siRNA). *Ache*-siRNA or *M1r*-siRNA (transfection with siRNA against *Ache*, or *M1r)*. MTT analysis (**A**), and *Ache* and *M1r* gene expression analysis (**B**). The mean ± SEM was obtained from data of three replicates of cultures performed three different times. Student’s *t*-test (*siRNA-M1R/AChE*-control wild-type cells or negative control comparisons) analyses were developed to determine statistically significant differences between treatments. *** *p* ≤ 0.001, significantly different from controls.

*Ache* knockdown or transfection with a negative control in SN56 cells did not affect cell viability, but the transfection with *M1r* siRNA decreased the cell viability (Figure 5A; t_(16)_ = 34.2, *p* < 0.0001) according to Student’s *t*-test statistical analysis. Transfection of SN56 cells with a negative control siRNA did not alter *Ache* and *M1r* gene expression (Figure 5B). Transfection of SN56 cells with *Ache* or *M1r* siRNA alone significantly decreased *Ache* (t_(10)_ = 35.0, *p* < 0.0001) or *M1r* (t_(10)_ = 38.9, *p* < 0.0001) gene expression (Figure 5B) according to Student’s *t*-test statistical analysis.

### 3.5. Analysis of PFOS Effects on SN56 Cell Viability and Activation of Caspases 3/7

**Figure 6:**
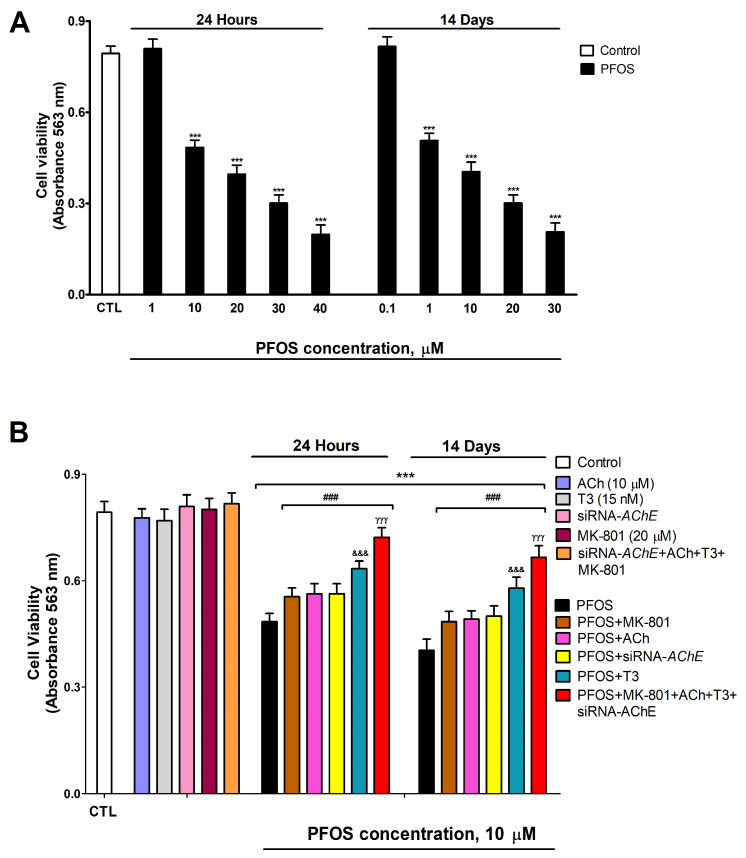
PFOS (1–40 µM) effects on cell viability (**A**). PFOS (10 µM) effect on the untransfected or *Ache* knockdown cells co-treated with or without MK-801 (20 µM), ACh (10 µM), and/or T3 (15 nM) after 1 or 14 days (**B**). The mean ± SEM was obtained from data of three replicates of cultures performed three different times. Student’s *t*-test (*siRNA-AChE cells*-control wild-type cells, ACh/T3/MK-801 treatment-control or siRNA-AChE cells treated with ACh-T3-MK-801-control comparisons), one-way (PFOS co-treatments-PFOS/vehicle treatment comparisons), and two-way (time-treatment comparisons) ANOVA analyses followed by the Tukey post hoc test were developed to determine statistically significant differences between treatments. *** *p* ≤ 0.001 compared to the control; ^###^ *p* ≤ 0.001 compared to PFOS treatment; ^&&&^ *p* ≤ 0.001 compared to PFOS treatment of *Ache*-silenced cells; ^γγγ^ p ≤ 0.001 compared to PFOS co-treatment with T3.

Neuronal viability was determined after 1- and 14-day PFOS treatment (0.1–40 µM) in SN56 cells. Two-way ANOVA analysis showed a significant time versus PFOS treatment interaction following single and repeated PFOS treatment in cell viability (F_(6,70)_ = 36.4, *p* < 0.0001). One-way ANOVA analysis showed a significant concentration-dependent PFOS treatment effect following single (cell viability: F_(6,35)_ = 488.1, *p* < 0.0001) and repeated (cell viability: F_(6,35)_ = 483.2, *p* < 0.0001) treatment. PFOS induced a statistically significant decrease in cell viability, compared with the control group, after one (starting at 10 µM concentration) and fourteen days (starting at 1 µM concentration) of treatment, which was greater as the concentration increased (Figure 6A). This reduction in cell viability was partially reversed after PFOS co-treatment with MK-801, ACh, or T3, or following PFOS treatment of *Ache*-silenced cells (Figure 6B). One-way ANOVA analysis showed a significant reversion of cell viability reduction after PFOS co-treatment/PFOS treatment of silenced cells compared to PFOS alone treatment following single (F_(5,53)_ = 90.1, *p* < 0.0001) and repeated (F_(5,53)_ = 83.5, *p* < 0.0001) treatment. Single MK-801, ACh or T3 treatment of wild-type cells, *Ache* silencing, or simultaneous MK-801, ACh, and T3 treatment of Ache-silenced cells did not alter cell viability (Figure 6B). The decrease in cell viability observed was not significantly different when comparing PFOS (10 µM) co-treatment with MK-801 or ACh, or PFOS treatment of *Ache*-silenced cells (Figure 6B). PFOS (10 µM) co-treatment with T3 led to a reduced significant decrease in cell viability than that produced following PFOS (10 µM) co-treatment with ACh or with MK-801, or after treatment of *Ache* knockdown cells (Figure 6B). PFOS (10 µM) concomitant co-treatment with MK-801, ACh, and T3 of *Ache* knockdown cells produced the highest reversion of the cell viability reduction observed following PFOS alone treatment of wild-type cells (Figure 6B). Results observed on control cells and vehicle treatment showed no significant variance.

**Figure 7:**
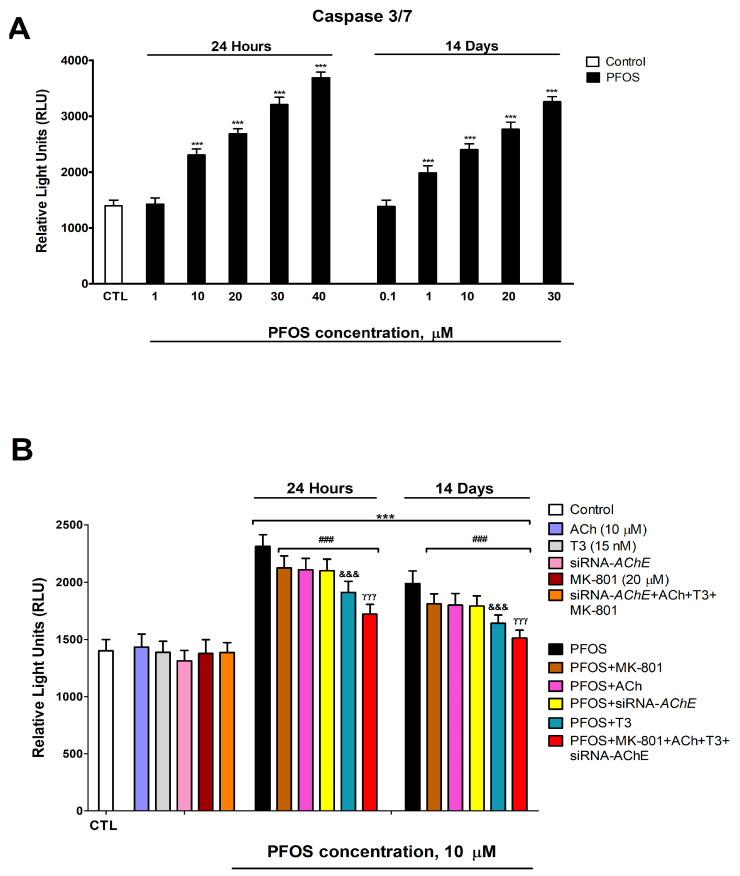
PFOS (1–40 µM) effects on caspases 3/7 activation (**A**). Analysis of caspases 3/7 activation after PFOS (10 µM) treatment of the wild-type or *Ache*-silenced cells co-treated with or without MK-801 (20 µM), ACh (10 µM), and/or T3 (15 nM) following 1 or 14 days (**B**). The mean ± SEM was obtained from data of three replicates of cultures performed three different times. Student’s *t*-test (*siRNA-AChE cells*-control wild-type cells, ACh/T3/MK-801 treatment-control or siRNA-AChE cells treated with ACh-T3-MK-801-control comparisons), one-way (PFOS co-treatments-PFOS/vehicle treatment comparisons), and two-way (time-treatment comparisons) ANOVA analyses followed by the Tukey post hoc test were developed to determine statistically significant differences between treatments. *** *p* ≤ 0.001 compared to the control; ^###^ *p* ≤ 0.001 compared to PFOS treatment; ^&&&^ *p* ≤ 0.001 compared to PFOS treatment of *Ache*-silenced cells; ^γγγ^ *p* ≤ 0.001 compared to PFOS co-treatment with T3.

Caspases 3/7 activation was determined after one- and fourteen-day PFOS treatment (0.1 µM–40 µM) in SN56 cells as an apoptosis marker. Two-way ANOVA analysis showed a significant time versus PFOS treatment interaction following single and repeated PFOS treatment in caspase activation (F_(6,70)_ = 13.4, *p* < 0.0001). One-way ANOVA analysis showed a significant concentration-dependent PFOS treatment effect following single (caspase activation: F_(6,35)_ = 472.5, *p* < 0.0001) and repeated (caspase activation: F_(6,35)_ = 284.3, *p* < 0.0001) treatment. PFOS induced a statistically significant increase in caspases 3/7 activation, compared with the control group, after one (starting at 10 µM concentration) and fourteen days (starting at 1 µM concentration) of treatment, which was greater as the concentration increased (Figure 7A). This increment of caspases 3/7 activation was partially reversed after PFOS co-treatment with MK-801, ACh, or T3, or following PFOS treatment of *Ache*-silenced cells (Figure 7B). One-way ANOVA analysis showed a significant reversion of caspase activation after PFOS co-treatment/PFOS treatment of silenced cells compared to PFOS alone treatment following single (F_(5,53)_ = 39.0, *p* < 0.0001) and repeated (F_(5,53)_ = 30.2, *p* < 0.0001) treatment. Single MK-801, ACh or T3 treatment, *Ache* silencing, or simultaneous MK-801, ACh, and T3 treatment of *Ache*-silenced cells did not induce caspases 3/7 activation (Figure 7B). The increase in the caspases 3/7 activation observed was not significantly different when comparing PFOS (10 µM) co-treatment with MK-801 or ACh, or after PFOS treatment of *Ache*-silenced cells (Figure 7B). PFOS (10 µM) co-treatment with T3 led to a reduced significant increase in caspases 3/7 activation than that produced following PFOS (10 µM) co-treatment with ACh or with MK-801, or after PFOS treatment of *Ache* knockdown cells (Figure 7B). PFOS (10 µM) simultaneous co-treatment with MK-801, ACh, and T3 of *Ache*-silenced cells produced the highest reversion of the caspases 3/7 activation observed following PFOS alone treatment of wild-type cells (Figure 7B). Cell viability reduction and activation of caspases, both after single and repeated treatment was graduated in the following decreasing order: PFOS > PFOS + MK-801 = PFOS + ACh = PFOS + SiRNA AChE > PFOS + T3 > PFOS + MK-801 + ACh + SiRNA AChE + T3. Results observed on control cells and vehicle treatment showed no significant variance. Caspase 3/7 activation data corroborate cell viability results and suggest that PFOS-induced cell death is mediated through apoptosis.

## 4. Discussion

TRα activity and levels were decreased and D3 levels were increased following one (starting at 10 µM) and fourteen days (starting at 1 µM) of PFOS treatment. This effect increased following the treatment concentration level and was greater after repeated treatment, showing that PFOS disrupts TH function through altering TRα activity and TH metabolism, which could lead to the cognition dysfunction reported after PFOS exposure. PFOS was reported to directly bind to TRα and present agonistic activity at low concentrations (from 5 µM), but antagonist activity at higher concentrations (from 60 µM) after a single treatment in GH3 rat pituitary cancer cells [55], supporting our results. PFOS single treatment was reported to upregulate TRα in zebrafish larvae [78], showing the capacity of PFOS to regulate TRα expression and the differences in the effect observed could be related to differences between species, protocol performed, concentrations, and exposure time employed, and age of exposure. PFOS was reported to upregulate D3 expression after repeated exposure in adult male rat liver [53], supporting our results. PFOS reduction in TRα activity seems to be mediated by the reduction in TRα levels observed and by direct antagonist activity, as previously described, and probably by a reduction in the levels of T3 due to the increase in D3 levels, contributing to the reduction in TRα activity observed.

ACh, ChAT, and M1R levels, ChAT and AChE activity, and selective M1R pirenzepine binding were decreased after PFOS treatment for one (starting at 10 µM) and fourteen days (starting at 1 µM), and these effects increased following the treatment concentration level and were greater after repeated treatment, showing that PFOS disrupts cholinergic transmission. To our knowledge, the PFOS effect on M1R levels and binding was not studied previously. PFOS was described to decrease muscarinic receptor density in Greenland polar bears’ brain regions [16] and downregulate M1R expression in the nervous system of zebrafish embryos [14], supporting our data. PFOS was shown to increase ACh content in the nervous system of zebrafish embryos [14], or developmental northern leopard frogs [15], supporting that PFOS alters ACh content, and the differences with our results could be due to the differences between adult and developmental animals and differences between species, among other factors. ACh regulates cognitive functions and the decrease in its levels, as observed in AD, leads to cognitive decline [79]. M1R downregulation or blockage produces memory impairment [22,23]. Therefore, the observed cholinergic transmission disruption could mediate the cognition decline produced after PFOS treatment.

PFOS was shown to decrease AChE activity in developing zebrafish larvae [18], in Greenland polar bears brain regions [16], and planarians *Dugesia japonica* after repeated exposure [19], supporting our finding. However, PFOS was also reported to increase and decrease AChE activity in zebrafish depending on the sex, dose, and time of exposure [17], being necessary to determine the factors that could lead to these different effects on AChE activity in the brain. PFOS was described to decrease ChAT activity in the frontal cortex of adolescent rats exposed in utero to PFOS [20] and decrease its expression in developing zebrafish larvae [18], which points out that the reduction in the ChAT activity observed was produced by the reduction in its expression, not discarding a direct binding and inhibition of the enzyme, supporting our results. However, PFOS was also reported to increase and decrease ChAT activity in zebrafish depending on the sex, dose, and time of exposure [17], being necessary to determine the factors that could lead to these different effects. The reduction in the ACh content observed seems to be mediated by the reduction in the ChAT activity, which synthetizes ACh [80], since AChE inhibition should lead to an increase in ACh content, but we cannot discard that the transporters, which mediate its release and uptake, could be also altered.

T3 co-treatment with PFOS partially reversed the decreased ACh, ChAT, and M1R levels, ChAT and AChE activity inhibition, and M1R blockage observed after PFOS treatment alone, pointing out that PFOS disrupts TH actions, leading to cholinergic neurotransmission alteration. THs rule the cholinergic system [45], regulating ChAT expression [81,82], muscarinic receptors expression and ligand affinity [48], AChE activity and ACh release [46]. TH deficiency was shown to decrease ACh content, M1R levels, ChAT and AChE activity, and M1R affinity in BFCN and T3 supplementation reversed these effects [24], corroborating our findings. However, the reversion produced after T3 co-treatment with PFOS was incomplete, suggesting additional mechanisms could be involved. Insulin regulates ChAT activity [83], ACh content [84], M1R levels and affinity [85], and AChE activity [86]. PFOS was reported to induce insulin resistance [87]. Therefore, this mechanism could also contribute to the cholinergic transmission disruption observed.

AChE-S and -R variants expression was also increased after PFOS treatment for one (starting at 10 µM) and fourteen days (starting at 1 µM), and this effect was greater as the concentration increased and after repeated treatment. To our knowledge, this is the first time the alteration induced by PFOS on AChE variants was explored. PFOS was described to increase AChE expression in the nervous system of zebrafish embryos [14,18] and decrease AChE expression in mice cortex after one-day exposure [79]. PFOS was also reported to increase and decrease AChE activity in zebrafish depending on the sex, dose, and time of exposure [17], showing the ability of PFOS to regulate the expression of AChE and supporting our results. M1R blockage was reported to upregulate AChE variants in BF SN56 cholinergic neurons [26]. Thus, PFOS could mediate this effect through this mechanism. T3 co-treatment with PFOS reverted partially the upregulation of AChE variants observed after PFOS treatment, but as this reversion is incomplete this suggests that additional mechanisms could be involved. THs were described to upregulate AChE expression in neuro-2A cells [47,88]. T3 deficiency was shown to increase AChE variants in BFCN, and its supplementation reversed, in part, this upregulation [24], supporting our findings. Insulin regulates AChE expression [86], so insulin disruption could also contribute to this effect.

Moreover, glutamate level and glutaminase activity were increased, and NMDAR1 level was decreased after PFOS treatment for one (starting at 10 µM) and fourteen days (starting at 1 µM), and these effects were greater as the concentration increased and were greater after repeated treatment. M1R silencing or T3 co-treatment with PFOS reverted partially these effects induced by PFOS alone treatment, showing that PFOS disrupts glutamatergic transmission through TH action dysfunction, mediated by M1R blockage. AChE-S was associated with glutamatergic transmission disruption [89], so the AChE-S overexpression induced through thyroid dysfunction could also mediate this effect. To our knowledge, this is the first study that shows PFOS effect on glutaminase activity. Glutaminase mediates glutamate synthesis [90], so the increased activity observed could mediate the increase in the glutamate levels observed, although we cannot discard a contribution of an alteration of glutamate metabolism and release. PFOS increases glutamate levels and induces excitotoxicity in rat primary cerebellar granule neurons through activation of NMDAR following 24 h of treatment [36]. PFOS was shown to increase glutamate levels in adult mice hippocampal neurons after repeated exposure [37,38]. Further, PFOS lactational exposure increases glutamate levels in mice’s adult hippocampus [91], supporting our findings. However, was also described that PFOS repeated exposure decreases glutamate levels in developing Northern leopard frog and adolescent mice brains [15,92], which could be related to differences between species, the protocol used, since glutamate levels were determined in the whole brain, or due to the phase of development tested, being necessary further studies to clarify this issue. Prenatal and lactational PFOS exposure in rats upregulates NMDAR1 in the frontal cortex, suggesting that this effect was mediated through TR [61], showing PFOS ability to regulate NMDAR1 levels. The differences with our results could be due to the developmental phase in which the studies were performed.

M1R was shown to upregulate glutamate levels [33,34], and NMDAR expression and activity [35], supporting our findings. M1R antagonists block the NMDAR regulation of working memory in the frontal cortex of rhesus macaques, and M1R agonists revert NMDAR dysfunction, and the consequent cognitive disorders [93]. T3 regulates extracellular glutamate levels decreasing them [49]. Hypothyroidism increases glutamate levels in human cingulate cortex, but they are decreased in hyperthyroid patients [50]. However, hypothyroidism decreases glutamate release and glutaminase activity in the rat hippocampus and supplementation reduces these effects [51]. These discrepancies with our results could be due to differences between the in vitro and in vivo models used and the protocol performed. Hypothyroidism was shown to decrease NMDAR1 expression in the rat hippocampus [52], supporting our findings. PFOS’ mechanisms, in addition to the reduction in TH actions, could contribute to the effect observed. In this sense, insulin resistance decreases NMDAR1 expression [94], increases glutamate levels [95] in mice’s hippocampus, and increases glutaminase activity in humans’ liver [96]. Therefore, this mechanism could also contribute to glutamatergic transmission disruption and the cognitive decline observed.

Lastly, PFOS treatment for one (starting at 10 µM) and fourteen days (starting at 1 µM) produced cell death, which was likely induced through apoptosis, and this effect was greater as the concentration increased and was greater after repeated treatment. PFOS was reported to induce neurodegeneration of cholinergic neurons in the nematode Caenorhabditis elegans [13]. PFOS single treatment induces apoptosis in humane neuroblastoma SH-SY5Y cells starting at 50 µM, and in rat primary cerebellar granule neurons starting at 10 µM [2,3,63], and after repeated treatment in adult mice hippocampal neurons [38], supporting our data. MR1 silencing induced cell death, indicating that the M1R blockage and reduction in its levels induced by PFOS exposure mediate the cell death observed. PFOS co-treatment with MK-801, ACh, or T3 of wild-type cells or PFOS treatment of AChE-silenced cells produced a reduced decrease in neuronal viability and increase in neuronal death than that produced following PFOS single treatment, suggesting that these mechanisms are involved in the neuronal death induced. Cell death, both after single and repeated treatment was graduated in the following decreasing order: PFOS > PFOS + MK-801 = PFOS + ACh = PFOS + SiRNA-AChE > PFOS + T3 > PFOS + MK-801 + ACh + SiRNA-AChE + T3, showing that the higher reversion was produced with PFOS + MK-801 + ACh + SiRNA-AChE + T3 co-treatment.

ACh regulates cognitive function and participates in the maintenance of cell viability [21]. M1R mediates BFCN viability and cognitive function, and its downregulation or blockage, as observed in AD, produces memory impairment [22,23], and BFCN loss [24,25]. Glutamate is necessary to maintain neuronal viability and cognitive functions [29,30,31]. However, elevated glutamate levels lead to neurodegeneration and cognitive decline [29,30,31]. NMDARs are necessary to maintain synaptic plasticity, cognitive function, and neuronal viability, but their overactivation leads to cognitive dysfunction [30], and BFCN loss [90]. AChE-S overexpression produces apoptotic cell death [97,98,99], and AChE silencing avoids apoptosis [99,100], suggesting that AChE-S upregulation induces, in part, the cell death observed. Hypothyroidism induces neurodegeneration in rat hippocampal neurons, and NMDAR antagonists revert this effect [101]. Hypothyroidism was also described to induce BFCN loss and T3 supplementation reversed this effect [102]. All these pieces of information support the results obtained.

Simultaneous PFOS co-treatment with MK-801, ACh, and T3 of AChE-silenced cells led to the lowest reduction in cell death, compared to that produced on wild-type SN56 after PFOS alone treatment, suggesting that further mechanisms may contribute to the cell death observed. PFOS was shown to induce apoptosis in SH-SY5Y cells and rat primary cerebellar granule neurons, through oxidative stress generation [2,3,63]. BFCN are especially sensitive to oxidative stress, leading to cell death [102,103,104]. Developmental PFOS exposure produces tau hyperphosphorylation (p-Tau) and Aβ protein aggregation in adult rats [105] (Zhang et al., 2016). Aβ and p-Tau protein accumulation was reported to induce BFCN death [102,103]. PFOS induces insulin resistance, which was reported to lead to hippocampal neurodegeneration [106], and BFCN loss [103]. Therefore, these mechanisms may also mediate the cell death observed.

## 5. Conclusions

In summary, after one (starting at 10 µM) and fourteen days (starting at 1 µM) treatments, PFOS resulted in TH activity dysfunction that led to cholinergic transmission disruption, mediated by ChAT inhibition and M1R reduced expression and activation; and glutamatergic neurotransmission disruption induced by glutaminase activity induction and decreased NMDAR1 levels, which were also disrupted through M1R signaling dysfunction. Additionally, PFOS-induced cell death, probably through apoptosis, is mediated, in part, by NMDAR overactivation, AChE-S overexpression, M1R signaling blockage, and thyroid signaling disruption. Additional research should be performed to analyze the mechanisms through which PFOS produces the effects on BFCN mentioned above and confirm whether they are reproduced in vivo and whether they mediate the cognitive disorder observed, which would consolidate the knowledge of PFOS mechanisms that induce BFNC neurodegeneration and may lead to cognitive dysfunction. The relevance of our data is to show novel mechanisms through which PFOS produces cholinergic and glutamatergic neurotransmission dysfunction and neurodegeneration of BFCN, providing novel therapeutic tools to treat these effects and possibly provide the mechanisms through which PFOS produces cognitive dysfunctions.

## Figures and Tables

**Table 1:** Primers used for AChE variants quantitative real-time PCR analyses [73].

Abbreviation	Gene	Forward (F) and Reverse (R) Primers
*Ache-S*	*Acetylcholinesterase*	F-ctgaacctgaagcccttagag R-ccgcctcgtccagagtat
*Ache-R*	*Acetylcholinesterase*	F-gagcagggaatgcacaag R-ggggaggtaaagaagagag

## Data Availability

The data presented in this study are available on request from the corresponding author.

## Supplementary Materials

The following supporting information can be downloaded at: https://www.mdpi.com/article/10.3390/biomedicines12112441/s1, Table S1: Student’s *t*-test statistical analysis results of comparison of PFOS co-treatment with T3 with PFOS alone treatment; Table S2: Student’s *t*-test statistical analysis results of comparison of PFOS co-treatment with T3 with PFOS alone treatment; Table S3: Student’s *t*-test statistical analysis results of comparison of PFOS co-treatment with T3 with PFOS alone treatment.
