# Supplementary material for: Thyroid Hormone Neuroprotection Against Perfluorooctane Sulfonic Acid Cholinergic and Glutamatergic Disruption and Neurodegeneration Induction
Source: Biomedicines. 2024 Oct 24;12(11):2441. doi: 10.3390/biomedicines12112441 (PMC11591898; doi:10.3390/biomedicines12112441)
Supplement: Supplementary file 1 [file biomedicines-12-02441-s001.zip › biomedicines-3247059-supplementary.pdf]

Table S1. Student's t-test statistical analysis results of comparison of PFOS co-treatment with T3 with PFOS alone treatment.

Time	Treatment	ACh Content	AChE Activity	ChAT Activity	ChAT Levels
24 h	PFOS (10 μM)+ T3 (15 nM)	$t_{(10)}=24.3$, $p < 0.0001$	$t_{(10)}=6.3$, $p < 0.0001$	$t_{(10)}=5.9$, $p < 0.0001$	$t_{(10)}=5.2$, $p < 0.0001$
	PFOS (20 μM)+ T3 (15 nM)	$t_{(10)}=12.9$, $p < 0.0001$	$t_{(10)}=6.3$, $p < 0.0001$	$t_{(10)}=6.4$, $p < 0.0001$	$t_{(10)}=7.2$, $p < 0.0001$
	PFOS (30 μM)+ T3 (15 nM)	$t_{(10)}=7.6$, $p < 0.0001$	$t_{(10)}=6.9$, $p < 0.0001$	$t_{(10)}=7.9$, $p < 0.0001$	$t_{(10)}=7.1$, $p < 0.0001$
	PFOS (40 μM)+ T3 (15 nM)	$t_{(10)}=6.4$, $p < 0.0001$	$t_{(10)}=6.5$, $p < 0.0001$	$t_{(10)}=8.9$, $p < 0.0001$	$t_{(10)}=5.6$, $p < 0.0001$
14 Days	PFOS (1 μM)+ T3 (15 nM)	$t_{(10)}=6.8$, $p < 0.0001$	$t_{(10)}=6.6$, $p < 0.0001$	$t_{(10)}=6.5$, $p < 0.0001$	$t_{(10)}=6.3$, $p < 0.0001$
	PFOS (10 μM)+ T3 (15 nM)	$t_{(10)}=6.9$, $p < 0.0001$	$t_{(10)}=5.3$, $p < 0.0001$	$t_{(10)}=7$, $p < 0.0001$	$t_{(10)}=5.9$, $p < 0.0001$
	PFOS (20 μM)+ T3 (15 nM)	$t_{(1o)}=6.9$, $p < 0.0001$	$t_{(10)}=7.2$, $p < 0.0001$	$t_{(10)}=9$, $p < 0.0001$	$t_{(10)}=6.7$, $p < 0.0001$
	PFOS (30 μM)+ T3 (15 nM)	$t_{(10)}=6.7$, $p < 0.0001$	$t_{(10)}=5.8$, $p < 0.0001$	$t_{(10)}=8.5$, $p < 0.0001$	$t_{(10)}=6.2$, $p < 0.0001$

Table S2. Student's t-test statistical analysis results of comparison of PFOS co-treatment with T3 with PFOS alone treatment.

Time	Treatment	AChE-S Expression	AChE-R Expression	M1R Levels	M1R Binding
24 h	PFOS (10 μM)+ T3 (15 nM)	$t_{(10)}=5.7$, $p < 0.0001$	$t_{(10)}=6.9$, $p < 0.0001$	$t_{(10)}=6.7$, $p < 0.0001$	$t_{(10)}=6.5$, $p < 0.0001$
	PFOS (20 μM)+ T3 (15 nM)	$t_{(10)}=9.3$, $p < 0.0001$	$t_{(10)}=9$, $p < 0.0001$	$t_{(10)}=5.8$, $p < 0.0001$	$t_{(10)}=6.8$, $p < 0.0001$
	PFOS (30 μM)+ T3 (15 nM)	$t_{(10)}=10.7$, $p < 0.0001$	$t_{(10)}=9.2$, $p < 0.0001$	$t_{(10)}=7.1$, $p < 0.0001$	$t_{(10)}=6.9$, $p < 0.0001$
	PFOS (40 μM)+ T3 (15 nM)	$t_{(10)}=12.8$, $p < 0.0001$	$t_{(10)}=9$, $p < 0.0001$	$t_{(10)}=6.7$, $p < 0.0001$	$t_{(10)}=6.2$, $p < 0.0001$
14 Days	PFOS (1 μM)+ T3 (15 nM)	$t_{(10)}=9.3$, $p < 0.0001$	$t_{(10)}=6.7$, $p < 0.0001$	$t_{(10)}=6.6$, $p < 0.0001$	$t_{(10)}=6.7$, $p < 0.0001$
	PFOS (10 μM)+ T3 (15 nM)	$t_{(10)}=8.7$, $p < 0.0001$	$t_{(10)}=9.3$, $p < 0.0001$	$t_{(10)}=6.5$, $p < 0.0001$	$t_{(10)}=5.7$, $p < 0.0001$
	PFOS (20 μM)+ T3 (15 nM)	$t_{(10)}=9.7$, $p < 0.0001$	$t_{(10)}=9.6$, $p < 0.0001$	$t_{(10)}=6.8$, $p < 0.0001$	$t_{(10)}=7.5$, $p < 0.0001$
	PFOS (30 μM)+ T3 (15 nM)	$t_{(10)}=11.4$, $p < 0.0001$	$t_{(10)}=9.6$, $p < 0.0001$	$t_{(10)}=6.9$, $p < 0.0001$	$t_{(10)}=6.6$, $p < 0.0001$

Table S3. Student's t-test statistical analysis results of comparison of PFOS co-treatment with T3 with PFOS alone treatment.

Time	Treatment	Glutamate Content	Glutaminase Levels	Glutaminase Activity	NMDAR1 Levels
24 h	PFOS (10 μM)+ T3 (15 nM)	$t_{(10)}=5.9$, $p < 0.0001$	$t_{(10)}=6.1$, $p < 0.0001$	$t_{(10)}=6.2$, $p < 0.0001$	$t_{(10)}=5.3$, $p < 0.0001$
	PFOS (20 μM)+ T3 (15 nM)	$t_{(10)}=6.0$, $p < 0.0001$	$t_{(10)}=7.5$, $p < 0.0001$	$t_{(10)}=8.4$, $p < 0.0001$	$t_{(10)}=5.0$, $p < 0.0001$
	PFOS (30 μM)+ T3 (15 nM)	$t_{(10)}=6.9$, $p < 0.0001$	$t_{(10)}=7.5$, $p < 0.0001$	$t_{(10)}=6.2$, $p < 0.0001$	$t_{(10)}=6.1$, $p < 0.0001$
	PFOS (40 μM)+ T3 (15 nM)	$t_{(10)}=7.6$, $p < 0.0001$	$t_{(10)}=8.2$, $p < 0.0001$	$t_{(10)}=7.5$, $p < 0.0001$	$t_{(10)}=5.5$, $p < 0.0001$
14 Days	PFOS (1 μM)+ T3 (15 nM)	$t_{(10)}=6.7$, $p < 0.0001$	$t_{(10)}=8.2$, $p < 0.0001$	$t_{(10)}=6.9$, $p < 0.0001$	$t_{(10)}=5.8$, $p < 0.0001$
	PFOS (10 μM)+ T3 (15 nM)	$t_{(10)}=6.9$, $p < 0.0001$	$t_{(10)}=7.1$, $p < 0.0001$	$t_{(10)}=5.7$, $p < 0.0001$	$t_{(10)}=5.3$, $p < 0.0001$
	PFOS (20 μM)+ T3 (15 nM)	$t_{(10)}=6.7$, $p < 0.0001$	$t_{(10)}=7.7$, $p < 0.0001$	$t_{(10)}=6.8$, $p < 0.0001$	$t_{(10)}=5.7$, $p < 0.0001$
	PFOS (30 μM)+ T3 (15 nM)	$t_{(10)}=6.9$, $p < 0.0001$	$t_{(10)}=7.6$, $p < 0.0001$	$t_{(10)}=7.1$, $p < 0.0001$	$t_{(10)}=5.1$, $p < 0.0001$
